# Changes in Circulating B Cell Subsets Associated with Aging and Acute SIV Infection in Rhesus Macaques

**DOI:** 10.1371/journal.pone.0170154

**Published:** 2017-01-17

**Authors:** W. L. William Chang, Denise F. Gonzalez, Hung T. Kieu, Luis D. Castillo, Ilhem Messaoudi, Xiaoying Shen, Georgia D. Tomaras, Barbara L. Shacklett, Peter A. Barry, Ellen E. Sparger

**Affiliations:** 1 Center for Comparative Medicine, University of California Davis, Davis, California, United States of America; 2 Department of Medicine and Epidemiology, School of Veterinary Medicine, University of California Davis, Davis, California, United States of America; 3 Department of Biomedical Sciences, School of Medicine, University of California Riverside, Riverside, California, United States of America; 4 Duke Human Vaccine Institute, Duke University Medical Center, Durham, North Carolina, United States of America; 5 Department of Medical Microbiology and Immunology, School of Medicine, University of California Davis, Davis, California, United States of America; 6 Division of Infectious Diseases, Department of Medicine, School of Medicine, University of California Davis, Davis, California, United States of America; 7 Department of Pathology and Laboratory Medicine, School of Medicine, University of California Davis, Davis, California, United States of America; 8 California National Primate Research Center, University of California Davis, Davis, California, United States of America; Emory University School of Medicine, UNITED STATES

## Abstract

Aging and certain viral infections can negatively impact humoral responses in humans. To further develop the nonhuman primate (NHP) model for investigating B cell dynamics in human aging and infectious disease, a flow cytometric panel was developed to characterize circulating rhesus B cell subsets. Significant differences between human and macaque B cells included the proportions of cells within IgD^+^ and switched memory populations and a prominent CD21^-^CD27^+^ unswitched memory population detected only in macaques. We then utilized the expanded panel to analyze B cell alterations associated with aging and acute simian immunodeficiency virus (SIV) infection in the NHP model. In the aging study, distinct patterns of B cell subset frequencies were observed for macaques aged one to five years compared to those between ages 5 and 30 years. In the SIV infection study, B cell frequencies and absolute number were dramatically reduced following acute infection, but recovered within four weeks of infection. Thereafter, the frequencies of activated memory B cells progressively increased; these were significantly correlated with the magnitude of SIV-specific IgG responses, and coincided with impaired maturation of anti-SIV antibody avidity, as previously reported for HIV-1 infection. These observations further validate the NHP model for investigation of mechanisms responsible for B cells alterations associated with immunosenescence and infectious disease.

## Introduction

An understanding of B cell biology and development is critical to characterizing the humoral immune response. B cells are lymphocytes derived from bone marrow lymphoid progenitor cells. Mature, naïve B cells migrate to lymphoid tissues, where they may be exposed to antigen and subsequently undergo differentiation and maturation into plasma cells or memory B cells. Plasma cells are long-lived antibody-secreting cells that localize predominantly within the bone marrow, whereas memory and naïve B cells circulate between blood and tissues. As the key component of the humoral immune response, antibodies play a significant role in the control of a wide variety of pathogens, and also contribute to the pathogenesis of certain autoimmune diseases [1]. However, B cell function and the humoral response may become perturbed or dysregulated by certain host conditions including chronic infection with pathogens such as herpes viruses [2–4] that establish lifelong persistence, or agents such as human immunodeficiency virus (HIV)-1 targeting immune response cells (e.g., CD4^+^ T cells) that directly interact with B cells [5–9]. Another host factor with significant impact on B cell function and the humoral response is deleterious aging of the immune system that is referred to as immunosenescence [10–12]. Investigation of mechanisms by which these various host conditions (e.g., aging, infection) perturb B cell function will require animal models that closely resemble the human host.

Much of the basic knowledge regarding B cell biology has been derived from laboratory mouse models. However, there are significant differences between human and murine B cells, which limit the usefulness of these models for elucidating human B cell function [11, 13, 14]. These differences point to a critical need for animal models that more closely mimic human biology for characterizing mechanisms of B cell-related diseases and identifying appropriate targets for therapeutic modulation of the humoral response. Nonhuman primates (NHP) including rhesus macaques (*Macaca mulatta*) infected with simian immunodeficiency virus (SIV) have been used extensively as animal models for testing of antiviral therapeutics and HIV-1 vaccine development [15]. Use of the NHP model has resulted in multiple reports characterizing B cell memory subsets and B cell functional assays for rhesus macaques [16–23]. However, many different flow cytometric panels have been utilized by different laboratories, and there has been no consensus regarding the most appropriate way to identify different B cell memory subsets in NHP. Similarly, studies investigating human B cell populations have also relied on a wide range of marker combinations, without apparent consensus [24, 25].

Therefore, one goal of studies described herein was to develop a flow cytometry staining panel with a combination of B cell markers that would not only identify naïve and memory cells, but would also distinguish multiple memory subsets and facilitate their comparison with analogous subsets in humans. This analysis was then applied to blood samples from healthy rhesus macaques in order to investigate changes in B cell subset frequencies associated with the aging process. The B cell staining panel was also used in conjunction with other assays to characterize circulating B cell subset alterations during acute and early chronic infection with pathogenic SIVmac251. Lastly, a preliminary comparison of B cell subset frequencies between healthy rhesus macaques and healthy human donors was performed using our expanded B cell staining panel. Our findings revealed important similarities between circulating B cell subsets in humans and rhesus macaques, as well as changes in these subsets with aging and acute viral infection in the NHP model.

## Materials and Methods

### Ethics statement

All studies conducted at the California National Primate Research Center (CNPRC) were approved in advance by the University of California Davis (UC Davis) Institutional Animal Care and Use Committee (IACUC; approval numbers 15951, 17606, and 17880). UC Davis has an Animal Welfare Assurance on file with the National Institutes of Health Office of Laboratory Animal Welfare (OLAW), and is fully accredited by the Association for the Assessment and Accreditation of Laboratory Animal Care, International (AAALAC). Studies conducted at the Oregon National Primate Research Center (ONPRC) were approved by the ONPRC IACUC (approval numbers 0456, 0785, and 0492) and conducted under strict accordance with the recommendations outlined in the Guide for the Care and Use of Laboratory Animals of the National Institute of Health OLAW and the United States Department of Agriculture. The ONPRC has been accredited by the American Association for Accreditation of Laboratory Animal Care since 1974.

SIV infection of susceptible macaques may produce a progressive fatal immunodeficiency disease characterized by hematologic abnormalities, lymphocyte depletion, diarrhea, weight loss and cachexia, and infection with opportunistic pathogens. In the course of study, situations may arise and clinical veterinary staff will determine if euthanasia is indicated before the planned endpoint following the Guidelines for Humane Euthanasia of Animals on Projects (GHEAP) at the CNPRC. GHEAP provides guidelines for selecting an endpoint that reduces animal pain and/or distress, while still meeting research objectives. All possible efforts are made to minimize pain and discomfort associated with SIV-associated disease. Analgesics (buprenorphine) may be given intramuscularly (IM) at the dosing range of 0.01 − 0.03 mg/kg body weight three times a day (TID) to minimize pain and discomfort at the discretion of the CNPRC veterinary staff and nutritional supplements may be administered.

### Experimental animals and samples

Two cohorts of animals at the CNPRC were used for these studies. One group of rhesus macaques (*n* = 44; see S1 Table for breakdown of age groupings), male and female, ranging in age from 1 − 5 years from the Specific Pathogen Free (SPF) colony, were used as blood donors for development of an expanded B cell staining panel and investigation of changes in circulating B cell subsets over ages 1 − 5 years. This SPF animal cohort was maintained as free of infection with SIV, type D retrovirus, simian T-cell lymphotropic virus type 1, simian foamy virus, herpes B virus (B virus), and rhesus cytomegalovirus (RhCMV). A second group of age-matched naïve female SPF rhesus macaques (*n* = 6, age of 2.5 − 3 yrs) was also used for a study involving primary infection with RhCMV for over six months, followed by subsequent infection with SIVmac251. Animals were administered 10 mg/kg body weight ketamine-HCl (Parke-Davis, Morris Plains, NJ, USA) IM when necessary for immobilization. Buprenorphine were administered IM at the discretion of the CNPRC veterinary staff to minimize pain and discomfort at the dosing range of 0.01 − 0.03 mg/kg body weight TID when necessary. Healthy donor macaques used for development and testing of the B cell staining panel were housed in outdoor or indoor housing, and were free of overt signs of disease. Animals involved in the RhCMV and SIV infection study were maintained at the CNRPC in cages with 4 square feet of floor space, or 6 square feet if over 10 kg, with fixed perch bars in a temperature-controlled BSL-2+ vivarium with continuous monitoring of temperature and humidity. Compatible animals were paired continuously or intermittently (separated at night) whenever possible. All animals had visual and auditory access to other macaques 24 hours per day. All animals were fed a balanced commercial macaque chow (Purina Mills, Gray Summit, MO) twice daily and fresh produce twice weekly, with free access to water 24 hours per day. Supplemental food was provided when clinically indicated. Environmental enrichment was provided daily, including manipulanda (forage boards, mirrors, puzzle feeders) and novel foodstuffs. Blood samples collected from all animals were processed for plasma and peripheral blood mononuclear cells (PBMC) by Accu Paque gradient centrifugation (Accurate Chemical & Scientific Corp., Westbury, NY) and cryopreserved at -80°C (plasma) or in liquid nitrogen (PBMC) for subsequent assessments.

A third cohort of animals, including conventionally raised (*i*.*e*., non-SPF) healthy rhesus macaques (*n* = 275; see S1 Table), male and female, of Indian origin bred, and ranging in age from 2 − 30 years from the ONPRC, were utilized for examination of circulating B cell subsets associated with aging. Animals used in this study were housed in indoor small group housing, and were free of overt signs of disease (neoplasms, acute infections, severe arthritis or wasting disease) and were maintained as the ONPRC. All animals were housed in quadrant cages (0.8 × 0.8 × 0.9 m) with constant temperature (20 − 22°C), humidity (65%) and a 11-h light cycle with visual, auditory and olfactory contact with other conspecifics. The barrier between monkeys housed side-by-side was removed for 2h/weekday and the monkeys shared the expanded housing cage. All animals were fed a balanced commercial macaque chow twice daily with occasional seasonal vegetables and fruit for enrichment and free access to water 24 hours per day. The animals had access to environmental enrichments like toys. All procedures were performed in the presence of qualified veterinary staff and all efforts were made to minimize animal suffering by use of analgesics administered at the discretion of the ONPRC veterinary staff. Sampling procedures for this cohort included anesthesia with 10 mg/kg body weight ketamine-HCl IM and were described previously [26].

Leukocyte-enriched buffy coats from healthy human donors (*n* = 12; see S2 Table for description of human donors by age and gender) were obtained from the Stanford Blood Center (Mountain View, CA). Human PBMC were isolated by Ficoll Paque gradient centrifugation (GE Healthcare, Pittsburgh, PA) and cryopreserved in liquid nitrogen until use.

### RhCMV and SIV infection of rhesus macaques

Age-matched naïve female rhesus macaques (*n* = 6, age of 2.5 − 3 yrs) were inoculated with a molecularly cloned variant of RhCMV, either RhCMV68-1 or RhCMVΔUL111A [27, 28], by a combination of subcutaneous inoculation (1 × 10^4^ PFU) and oral exposure (1 × 10^5^ PFU) applied to the buccal pouch and sublingual region. RhCMV virus stocks were prepared as previously described [28]. Animals were monitored for 28 weeks and subsequently received multiple low dose SIV challenge by the intravaginal (IVAG) route using protocols and a SIVmac251 virus stock as previously described [29, 30]. Animals were tested for plasma viremia for two consecutive weeks, or weekly until all animals tested positive for plasma SIV RNA. Peripheral blood was collected at two to four week intervals after confirmed infection for assay of virus-specific immune responses and immune cell subset analysis. All animals were carefully monitored daily for adverse effects associated with SIV infection to spare the animals of prolonged or unnecessary pain and discomfort. Animals were euthanized by overdose with pentobarbital (120 mg/kg) delivered intravenously at the termination of the study (within 28 weeks after challenge).

### Phenotypic analysis by flow cytometry

Multiparameter flow cytometry was performed using a BD FACSFortessa cell sorter operated by BD FACSDiva software (BD Biosciences, San Jose, CA). PBMC from rhesus macaques or human donors were stained with directly conjugated monoclonal antibodies against human (rhesus macaque cross-reactive) CD3 (clone SP34-2), CD20 (clone L27), CD21 (clone B-ly4), CD27 (clone M-T7271), IgM (clone G20-127), and IgG (clone G18-145) (all purchased from BD Biosciences) or directly conjugated polyclonal goat antibodies against human (rhesus macaque cross-reactive) IgD and IgA (both purchased from Southern Biotech, Birmingham, AL). Staining for intracellular immunoglobulins was performed with the Fixation/Permeabilization solution kit (BD Biosciences) according to the manufacturer’s instructions after surface staining with the appropriate antibodies using protocols previously described [28]. Data were analyzed and illustrated using FlowJo software (Tree Star Inc., Ashland, OR). The FACS data generated from post SIV infection samples can be found at FlowRepository (ID: FR-FCM-ZZVV, FR-FCM-ZYZZ, and FR-FCM-ZYZY).

### SIV RNA quantitation

Viral RNA was isolated from plasma samples using QIAamp Viral RNA Mini Kit (Qiagen, Valencia, CA) and viral RNA concentration was measured using a real-time reverse transcriptase TaqMan qPCR assay with a sensitivity limit of 50 copies of SIV RNA per mL of plasma as previously described [31].

### Antibody ELISAs and avidity assay

The binding IgG titers for RhCMV antigens and SIV Gag p27 were quantified by in-house ELISAs. Microtiter plates (Nunc MaxiSorp; Thermo Fisher Scientific, Waltham, MA) were coated with 250 ng/well purified RhCMV virions (heat-inactivated) or with 50 ng/well recombinant SIVmac251 Gag p27 protein (ImmunoDX, Woburn, MA) in coating buffer (0.375% sodium bicarbonate/Hanks buffered salt solution) at 4°C overnight. Plates were then washed six times with PBS/0.05% Tween 20 (PBST), and subsequently blocked with PBS/1% BSA at 25°C for 2h. All plasma samples and control standards were diluted in PBST/0.5% BSA. Diluted samples were added to duplicate wells and incubated for 2h at 25°C. Plates were then washed six times with PBST, and bound antibodies were detected using HRP-conjugated goat anti-monkey IgG polyclonal antibodies (KPL, Gaithersburg, MD) and TMB substrate (Thermo Fisher Scientific). Each ELISA plate included an eight-point standard, prepared by making serial twofold dilutions of each standard plasma sample (1:200 to 1:25,600 for RhCMV standard; 1:1,600 to 1:204,800 for SIV Gag p27 standard), to generate a regression curve for converting OD_450_ values of samples to relative IgG units by a log-log model equation. All the samples were diluted to fit within the standard curve and results were reported as relative IgG units (at 1:400 dilution) after multiplying by the dilution factors.

Avidity assays were carried out by procedures similar to the antibody ELISA described above, except that the first wash after removing plasma was performed with PBST containing 6M urea. Detailed procedures and the formula for avidity index calculation were described previously [28].

### SIV Env-specific binding antibody multiplex assay

Plasma IgG binding to SIVmac239 Env gp130 was determined by custom SIV binding antibody multiplex assay (SIV-BAMA) as previously described [32, 33]. Purified IgG from a SIV-infected macaque (DBM5, a gift from M. Roederer, NIH Vaccine Research Center, Bethesda, MD) was used as the positive control and to track the SIVmac239 Env gp130 protein by Levy-Jennings plots using 21CFR Part 11 compliant software. Binding magnitudes of samples (at 1:1280 dilution) are reported as mean fluorescence intensity (MFI) values within the linear range of the assay.

### Statistical analysis

Statistical analysis and graphing was conducted with Prism software (GraphPad Software, Inc., San Diego, CA). *P* values less than 0.05 were considered significant. Comparisons of two groups were made with paired, two-tailed Student’s *t* tests, nonparametric Wilcoxon tests, or nonparametric Mann-Whitney tests. Comparisons of multiple groups were performed with Friedman nonparametric tests followed by Dunn’s multiple comparison tests. Correlation analyses were performed by Spearman’s rank tests.

## Results

### Development of staining panels for flow cytometric analysis of circulating B cells in rhesus macaques

**Fig 1:**
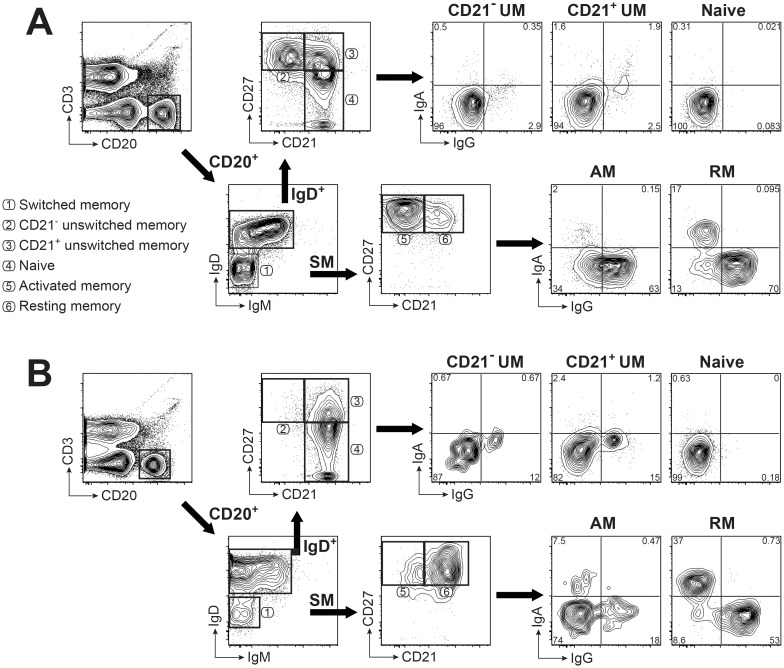
Gating strategy for identification of memory B cell subsets in rhesus macaque and human PBMC. B cell phenotyping panels with the same markers and gating strategy are illustrated for macaque (**A**) and human (**B**) PBMC. Shown are 5% contour plots with outliers of cells after gating on singlets, then lymphocytes, and exclusion of dead cells. B cells were identified by gating on CD3^-^ CD20^+^ cells. Naïve and unswitched memory (UM) B cells were defined using CD21 and CD27 after gating on IgD^+^ B cells. Activated memory (AM) and resting memory (RM) B cells were defined by using CD21 and CD27 in the IgD^-^ IgM^-^ switched memory (SM) B cell subset. Surface expression of IgG and IgA on each B cell subset is shown in the right panel. Numbers represent percentages of the respective cell populations among the total gated cells. Circled numbers associated with different gated populations identify the B cell subset within that gate as noted in the list of subsets.

A flow cytometric panel for phenotyping of circulating B cell subsets was optimized to examine changes in B cell memory phenotype associated with aging and infectious disease. This panel is based on surface markers CD3, CD20, IgD, IgM, CD27, and CD21 that are used to distinguish naïve (CD3^-^CD20^+^IgD^+^CD21^+^CD27^-^), unswitched memory (UM; CD3^-^CD20^+^IgD^+^CD21^+^CD27^+^ and CD3^-^CD20^+^IgD^+^CD21^-^CD27^+^), and switched memory (SM; CD3^-^CD20^+^IgD^-^IgM^-^) B cell subsets as previously described [24]. These subsets are shown by a scatterplot analysis of PBMC isolated from a representative rhesus macaque in Fig 1A. The SM B cell subset was further subdivided into resting memory (RM; CD21^+^CD27^+^), activated memory (AM; CD21^-^CD27^+^), and double negative memory (DN; CD21^-^CD27^-^) subsets [24, 34]. This rhesus macaque B cell staining panel differs from other frequently used panels that rely on either CD21 and CD27 [16–18, 21, 35] or IgD and CD27 [19, 24, 36–38], but not all three. Instead, our strategy uses IgD, CD21 and CD27 together; this approach has the advantage of distinguishing switched versus unswitched memory populations, while also differentiating resting versus activated memory subsets within the larger population of SM B cells (Fig 1A). A fourth memory subset, defined as either a IgD^-^CD21^lo/-^CD27^-^ DN cells or as a tissue-like memory (TLM) population (CD21^low/-^CD27^-^), may also be identified by this gating strategy but is not specifically illustrated in Fig 1. Frequencies of less than 5% for DN or TLM subsets within circulating B cells for macaques are comparable to frequencies reported for healthy humans [39, 40].

This same panel was also used to stain PBMC from healthy human donors for a comparison of memory B cell subset distribution in blood as demonstrated by a representative healthy donor in Fig 1B. Interestingly, representative staining of macaque and human circulating B cells revealed notable differences including a prominent CD21^-^CD27^+^ UM population and a reversed ratio of RM to AM frequencies in the SM B cell subset for a healthy young adult macaque, compared to a healthy human donor. A noticeable CD21^-^CD27^+^ UM population observed for macaque B cells was essentially absent in circulating human B cells (Fig 1) and has rarely been reported for macaques most likely due to limitations of frequently used B cell staining panels. Typically, UM B cells in blood have been considered as circulating splenic marginal zone (MZ) B cells [25, 41], referred to as either IgD^+^CD27^+^ or IgD^+^CD21^+^CD27^+^ with few if any reports of an IgD^+^CD21^-^CD27^+^ UM population in humans, although this population was previously described in macaques [16]. These findings suggest that gating strategies that include CD21 and CD27 alone without IgD (as shown in S1A Fig) will not distinguish CD27^+^ UM and SM subsets and will fail to reveal the CD21^-^CD27^+^ UM population observed for circulating B cells of rhesus macaques. Similarly, a B cell staining panel for rhesus macaque peripheral blood that includes only CD27 and IgD (as shown in S2A Fig) will also fail to distinguish a CD21^-^CD27^+^ UM subset and will not segregate SM B cells into RM and AM subsets. These findings corroborated the use of an analysis strategy that includes a combination of CD21, CD27 and IgD surface staining for precise identification of all B cell subsets.

### Increased intracellular staining for IgG follows development of activated memory B cells

**Fig 2:**
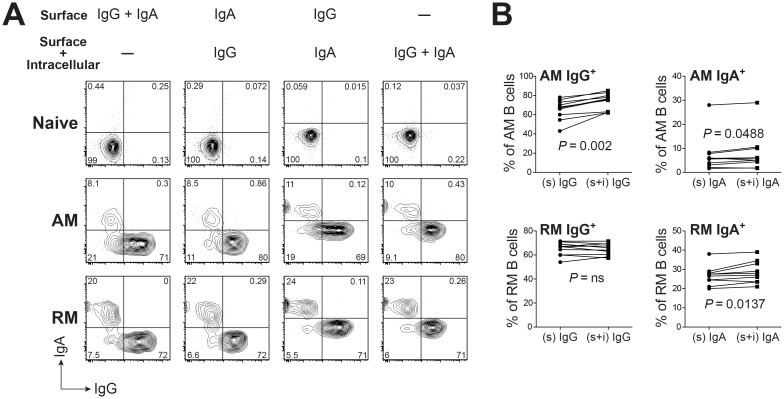
Characterization of isotype expressing memory B cells by combining surface and intracellular staining for IgG and IgA. (**A**) Representative FACS plots of circulating B cell subsets staining for IgG and IgA are shown. Numbers represent percentages of the respective cell populations among the total gated cells. Anti-IgG and/or Anti-IgA antibodies were added before (surface) or after (surface + intracellular) membrane permeablilization. (**B**) Comparison of IgG^+^ or IgA^+^ expression by AM or RM B cells by surface only (s) or surface plus intracellular (s + i) staining with isotype-specific antibodies. Statistical analyses were performed using nonparametric Wilcoxon tests. ns: not significant.

Staining for surface IgG and IgA was included to further characterize individual macaque B cell subsets by immunoglobulin isotypes and to confirm the gating strategy relative to the naïve and UM B cell populations that should show an absence or low frequency of IgG and IgA surface expression [42] (Fig 1). However, surface immunoglobulin staining patterns for SM subsets revealed a significant proportion of cells double negative for both IgA and IgG, particularly within the AM subset (Fig 1A, population 5). To test whether some immunoglobulin was intracellular and therefore not detected by the original staining protocol, the panel was modified to compare cell surface and intracellular staining for IgG and IgA expression by SM cells. Macaque PBMC were stained with memory markers and with IgG and IgA, at the cell surface and intracellularly (Fig 2A). Results revealed that intracellular staining for IgG and IgA detected a proportion of AM B cells that failed to express either isotype on the cell surface. Although the differences in expression were significant for both IgG and IgA in the AM subset (*P* = 0.002 and *P* = 0.0488 respectively), the effect was greater for IgG expression (Fig 2B). Intracellular staining resulted in a small increase in frequency of cells that express only IgA (*P* = 0.014) in the RM subset. The small populations within the AM and RM subsets that remained negative for IgA and IgG expression by both intracellular and surface staining were not further investigated. The predominant isotype expressed by both circulating memory subsets (RM and AM) was IgG (Fig 2B). Establishment of normal frequency ranges of circulating memory B cell subsets expressing IgG and IgA may be relevant for studying changes in immunoglobulin isotype expression with acute and chronic infections in rhesus macaques, as suggested by recent studies [43, 44].

### Differences in memory phenotype of circulating B cells in specific pathogen free rhesus macaques associated with early transitions in age

**Fig 3:**
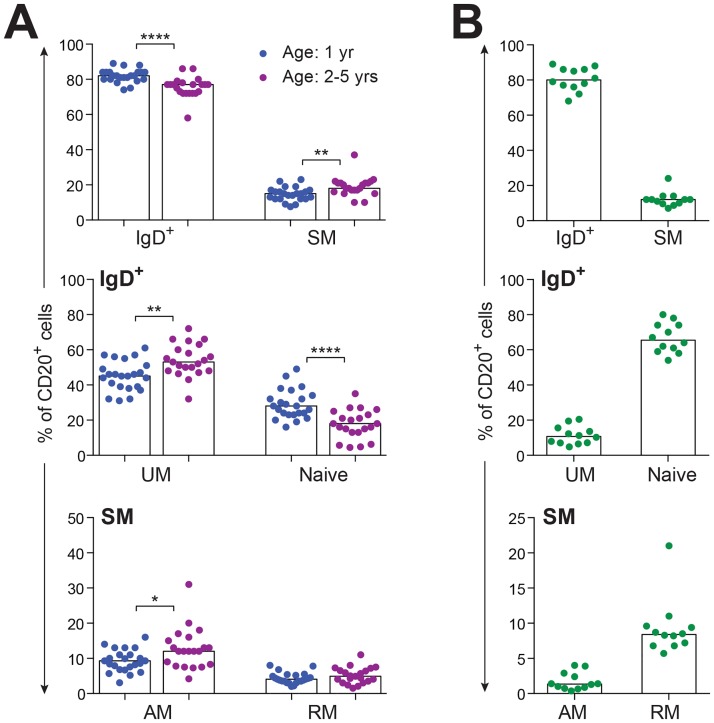
Distribution of B cell subsets in PBMC. (**A**) Frequencies of B cell subsets in peripheral blood of healthy SPF rhesus macaques (age 1 yr, *n* = 23; age 2 − 5 yrs, *n* = 21) are shown, with each data point representing an individual animal. (**B**) Frequencies of B cell subsets in peripheral blood of healthy human adults (*n* = 12) are shown, with each data point representing an individual donor. Bars in each graph indicate median frequencies. Statistical analyses between two age cohorts of SPF macaques were performed using nonparametric Mann-Whitney tests. Symbol: * *P* < 0.05; ** *P* < 0.01; **** *P* < 0.0001.

This expanded B cell staining panel was next used to interrogate PBMC isolated from healthy rhesus macaques raised under SPF conditions for naïve and memory B cell subsets. Macaques were classified by age to include one group (*n* = 23) at one year of age (young juveniles), and a second group (*n* = 21) at 2 − 5 years of age (older juveniles to young adults). Small but significant decreases (p < 0.0001) were noted in frequencies of circulating IgD^+^ and naïve B cell subsets as SPF macaques progressed from young juveniles to older juveniles and young adults (Fig 3A). Macaques in the older age group showed small but significant increases in frequencies of UM (*P* < 0.01), SM (*P* < 0.01), and AM (*P* < 0.05) B cell subsets compared to young juvenile SPF macaques (Fig 3A). Changes in frequency of the DN subset were not observed over this age range (data not shown) and were not anticipated based on previous studies revealing increases predominantly in elderly humans [3, 34, 39]. Collectively, these results suggested that rhesus macaques experience a loss of naïve B cells and small but significant increases in frequencies of circulating B cells transitioning from naïve to UM and SM memory subsets, including AM cells, by the time of young adulthood.

### Comparison of rhesus macaque and human circulating memory B cell subset frequencies

Based on preliminary observations (Fig 1), circulating B cell subset frequencies were analyzed in a cohort of healthy human adults (*n* = 12) (Fig 3B). This analysis revealed marked differences compared to B cell frequencies measured in the young adult macaque cohort (Fig 3A) and confirmed differences shown between macaque and human B cells in Fig 1. The naïve subset was the predominant circulating IgD^+^ B cell population in adult humans, while the IgD^+^ population in macaque donors revealed a higher proportion of UM B cells compared to naïve B cells (Fig 3A and 3B). Also, blood from adult humans contained a higher frequency of RM compared to AM B cells within the SM subset, with the opposite trend observed in young rhesus macaques. These differences suggest a higher proportion of circulating CD21^-^CD27^+^ B cells for young rhesus macaques compared to adult humans, which has not been previously appreciated.

Given that the gating strategy used for human B cells in our studies differed from other reports describing panels that included different combinations of B cell markers (e.g. CD10, CD24, CD38, IgD, CD21 and CD27 [24]), a second analysis of CD20^+^ cells including only CD21 and CD27 gating without IgD, was performed to determine the proportions of human naïve and memory B cell subsets (S1A Fig). A very similar distribution of CD20^+^ B cells between naïve, AM and RM subsets was observed when applying this gating strategy to human B cells (Fig 3B, S1B Fig) despite the absence of IgD gating. Taken together, results of these experiments revealed potential differences in B cell subsets between humans and rhesus macaques, that would not be detected by panels that rely only on IgD and CD27 as markers for memory subsets.

### Changes in memory phenotype of circulating B cell subsets over the lifespan of non-SPF rhesus macaques

To extend our investigation of aging and immunosenescence in circulating B cell subsets, an analysis was conducted in a separate, collaborating laboratory, on blood samples from a previously described, unique non-SPF rhesus macaque cohort (*n* = 275) [26, 38]. This cohort included animals ranging in ages from 2 to 30 years in age and housed within a different primate center (ONPRC). The cohort was divided into two groups: the first included macaques transitioning from juveniles to young adulthood (2 − 6 years) and the second included those transitioning from young to older adulthood (5 − 30 years). For this analysis, a different but frequently used B cell marker panel focusing on CD20, CD27, and IgD expression, without CD21, was utilized (S2A Fig) [45]. Circulating B cells were distinguished as CD3^-^CD20^+^ and next separated into SM (IgD^-^CD27^+^), UM (IgD^+^CD27^+^), naïve (IgD^+^CD27^-^) and, for the adult cohort only, DN (IgD^-^CD27^-^).

To verify that data from the two distinct flow cytometry panels were comparable for the B-cell subsets common to both panels, we re-analyzed the data previously described in Fig 3A, this time basing our analysis only on CD20, CD27 and IgD expression and excluding CD21. This new analysis, generated frequencies of SM, UM, and naïve subsets within the CD20^+^ population (S2B Fig). A comparison of results from this analysis with results from our optimized panel (Fig 1A) for SM, UM, and naïve subsets (Fig 3A), revealed comparable frequencies for the two different age groups regardless of the staining panel used. Statistically significant differences between the two different age groups for SM, UM, and naïve B cell subset frequencies (Fig 3A and S2B Fig) were also comparable for results generated by either gating strategy, with increases in UM and SM and decreases in the naïve subsets observed as SPF macaques transitioned from 1 year to 2 − 5 years of age.

Having established that the two flow cytometric panels gave comparable results for the cell subsets common to both analysis strategies, we next evaluated age-related changes for SM, UM, and naïve B cell subsets in the ONPRC non-SPF rhesus macaque cohort using the restricted staining panel. It is important to note that analysis of RM and AM populations within the SM subset was not possible for this cohort due to the absence of CD21 staining. However, multiple reports tracking B cell subset changes associated with aging in humans utilized this same restricted panel, based on IgD and CD27 staining for either CD20^+^ or CD19^+^ cells, which allows a comparison of our findings from other reports [10, 24, 46].

**Fig 4:**
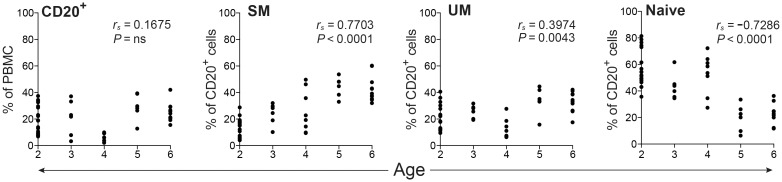
Frequencies of total CD20^+^ B cells and B cell subsets in juvenile and young adult non-SPF rhesus macaques. PBMC isolated from non-SPF macaques (age 2 − 6 yrs, *n* = 50) were assessed by FACS to determine the frequencies of CD20^+^ B cells, and SM, UM, and naïve, B cell subsets. Correlations between age and cell frequencies were determined by Spearman’s rank correlation.

Relationships between circulating B cell subset frequencies in the ONPRC macaque cohort were first examined over the ages of 2 to 6 years by correlation analyses (Fig 4). Similar to findings for SPF macaques represented in Fig 3A, an increasing frequency of SM (*P* < 0.0001) and a decreasing frequency of naïve B cells (*P* < 0.0001) were observed as non-SPF macaques transitioned from 2 to 6 years of age. A significant change in frequency of the UM subset (*P* = 0.0043) was also detected in this non-SPF cohort (ONPRC), although the correlation coefficient values for the regression analyses of this B cell subset revealed only a weak correlation, possibly due to the considerable variability in frequencies noted for the two year old age group. Overall, the trends for changes in naïve and memory subsets in the ONPRC cohort were comparable to those observed for the age-matched CNPRC cohort.

**Fig 5:**
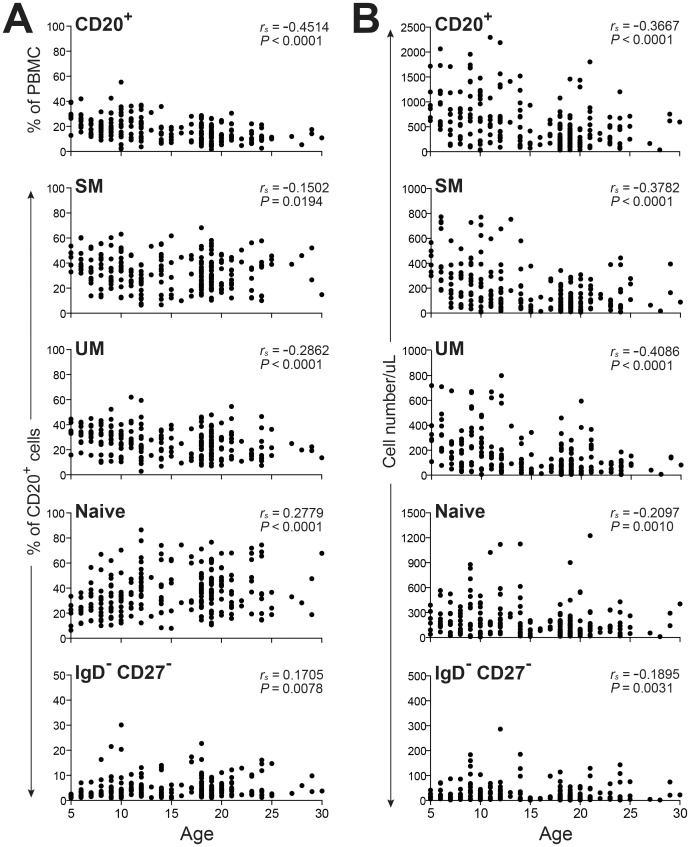
Frequencies and absolute cell numbers of circulating B cells in young adult, adult, and aged non-SPF rhesus macaques. PBMC isolated from non-SPF macaques distinguished as young adult and adult (age 5 − 18 yrs, *n* = 158) and aged (age >18 yrs, *n* = 84) were assessed by FACS to determine the frequencies (**A**) and absolute cell counts (**B**) of CD20^+^ B cells, and SM, UM, Naïve, IgD^-^CD27^-^ B cell subsets over time according to age. Correlations between age and cell frequencies were determined by Spearman’s rank correlation.

Analysis of macaques transitioning from young to elderly adults revealed that circulating cell counts decreased significantly for all B cell subsets including SM (*P* < 0.001), UM (*P* < 0.0001), naïve (*P* < 0.001) and DN (*P* < 0.0031) with increasing age (Fig 5B). This decreasing number of cells for all B cell subsets was most likely due to the progressive reduction of circulating total lymphocyte numbers (data not shown) in combination with a reduced frequency of CD20^+^ cells in PBMC (*P* < 0.001) (Fig 5A), resulting in a highly significant and progressive reduction of total CD20^+^ cell counts (*P* < 0.001) (Fig 5B). Regarding changes in B cell subset distribution, increasing frequencies for naïve (*P* < 0.0001) and DN (*P* = 0.0078) subsets and decreasing frequencies of UM (*P* < 0.0001) and SM (*P* = 0.0194) subsets were observed with aging of this macaque cohort (Fig 5A). However, the correlation coefficient values for these regression analyses of B cell subsets revealed only weak to very weak correlations, possibly due to the considerable variability in frequencies noted for each age group examined. Likewise age-related changes described for circulating B cell subsets in humans have been variable with both increased and decreased frequencies shown for SM, naïve and UM subsets reported. Standardization of staining panels for B cell subsets and normalization of healthy control cohorts may be required for developing definitive conclusions regarding the impact of aging on B cell memory subsets in humans as well as rhesus macaques.

### Circulating B cell counts and subsets are modulated during the acute phase of SIV infection

**Fig 6:**
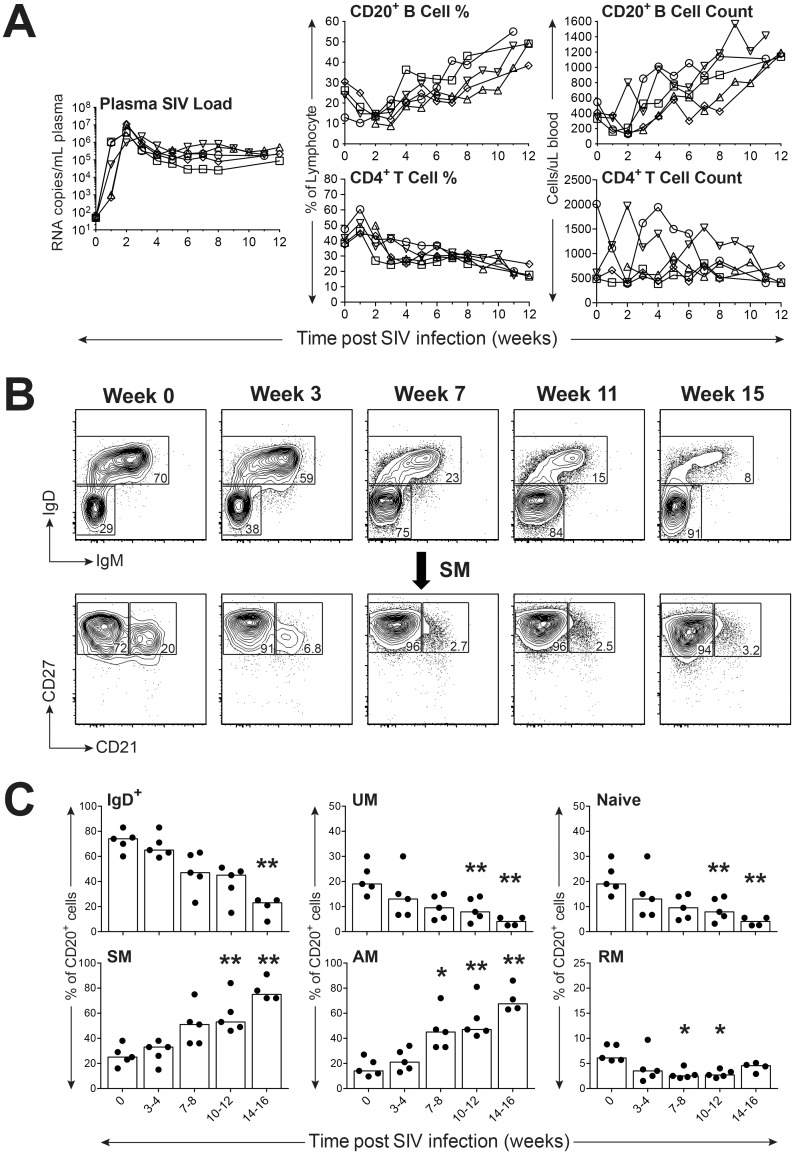
Impact of SIV infection on distribution of peripheral blood B cell subsets in rhesus macaques. (**A**) Measurement of viral loads, B and CD4^+^ T cell frequencies and counts during acute and early set point SIV infection are shown. (**B**) Representative FACS plots depict the progressive shift of circulating B cell subsets over the duration of SIV infection. (**C**) Frequencies of peripheral blood B cell subsets in typical progressors (*n* = 5) before and after SIV infection are shown with each data point representing an individual animal. Bars in each graph indicate median frequencies. Statistical analyses were performed using Friedman nonparametric tests followed by Dunn’s multiple comparison tests comparing each time point with the week 0 control. Symbol: * *P* < 0.05; ** *P* < 0.01.

Our expanded B cell staining panel was next used to examine B cell subsets in a group of six SPF adult female rhesus macaques experimentally infected with SIVmac251. Prior to infection with SIVmac251, these macaques were experimentally inoculated with a molecularly cloned variant of RhCMV, either RhCMV68-1 or RhCMVΔUL111A [27, 28]. Approximately six months post infection with RhCMV, macaques were infected with SIVmac251 by a low dose IVAG challenge and tested weekly to every other week for plasma SIV viral RNA loads, circulating B cell numbers, B cell subset changes, and anti-SIV and anti-RhCMV antibody responses over a 12 − 19 week period defined as acute to early chronic infection. All six macaques demonstrated infection based on detectable viral RNA in plasma after one to 12 IVAG challenges, with peak plasma virus loads ranging from 2.2 × 10^6^ to 1.1 × 10^7^ RNA copies per mL plasma by one to three weeks post infection (Fig 6A and S3A Fig). Five of the six infected macaques experienced a typical progressive infection characterized by a 10 − 100 fold decrease in peak plasma virus loads to set point loads ranging from 3 x 10^4^ to 5 x 10^5^ RNA copies per mL by five weeks after infection without evidence of AIDS-like clinical disease over a 20 week period (Fig 6A). However, one of the six infected macaques demonstrated an infection characteristic of a rapid progressor with high set point virus loads (between 3 − 8 × 10^6^ RNA copies per mL) throughout the initial 12 weeks post infection (S3A Fig). The five typical progressors demonstrated a precipitous but transient decrease in frequency of circulating CD20^+^ B cells and circulating CD20^+^ B cell counts within one to two weeks after infection. By three to four weeks post infection, both frequency and absolute counts of CD20^+^ B cells increased steadily throughout the remaining 12 week period of observation to reach B cell counts greater than those observed prior to infection by 12 weeks post infection (Fig 6A). Modulation of B cell subset frequencies in blood during acute SIV infection was also observed as shown by a longitudinal analysis with representative flow cytometry scatter plots from a representative animal (Fig 6B) and by median values for each subset frequency for the five normal progressor animals (Fig 6C). Progressive declines in the frequencies of IgD^+^, UM, and naïve subsets were observed over the 12 week period with significant differences apparent by 10 − 12 weeks post infection for the UM (*P* < 0.01) and naïve (*P* < 0.01) subsets (Fig 6C). In contrast, progressive increases were noted for frequencies of SM and AM subsets with a significant difference (*P* < 0.05) noted for the AM subset observed as early as 7 − 8 weeks after infection (Fig 6C). Small but significant decreases in frequency for the RM subset were also observed during the period of 7 − 12 weeks after infection (*P* < 0.05). Overall, B cell perturbations during the acute phase of typical progressor infections after low dose IVAG challenge included transient decreases in circulating B cell counts and B cell frequencies followed by progressively increasing B cell counts and frequencies that were also associated with increases of SM and AM B cell subsets.

### Changes in circulating B cell counts and subsets are associated with altered humoral immune responses during acute SIV infection

**Fig 7:**
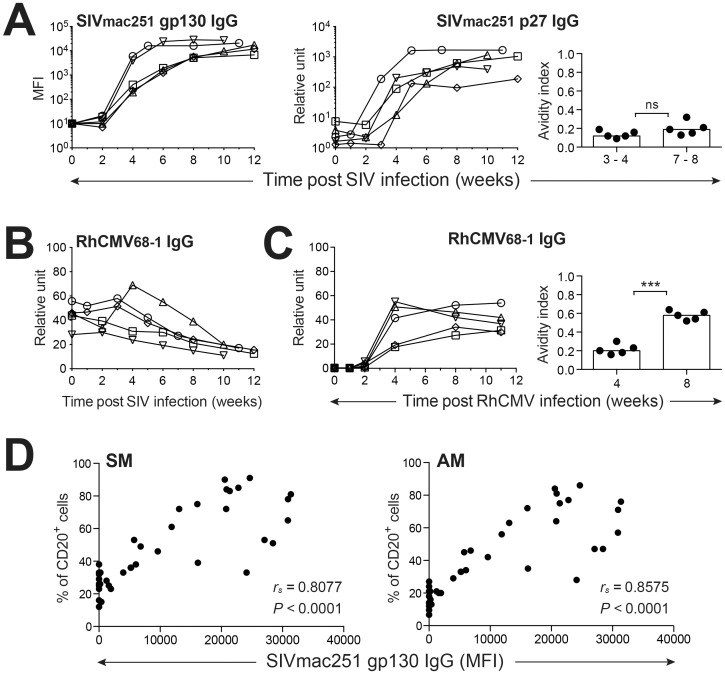
Comparison of antiviral antibody kinetics following SIV and RhCMV infection. (**A**) Plasma titers of anti-SIV gp130 and p27 IgG during SIV infection are shown. Changes in anti-SIV p27 avidity indices post SIV infection are shown on the right. (**B**) SIV infection impairs RhCMV-specific humoral immune responses. (**C**) Changes in plasma anti-RhCMV IgG titers and avidity indices are noted during primary RhCMV infection. (**D**) Correlation of SM and AM B cell frequencies in PBMC of SIV-infected macaques with plasma antibody titers of anti-SIV gp130 was determined. Statistical analyses of avidities indices were performed using Student’s *t* tests (paired, two-tailed). Correlation analyses were performed by Spearman’s rank tests. Symbol: *** *P* < 0.001. ns: not significant.

Emergence of IgG antibodies recognizing both SIVmac251 Gag p27 and Env gp130 proteins within three to four weeks after detectable infection was coincident with increasing circulating B cell counts and increased frequencies of SM and AM subsets for the typical SIV-infected progressors (Figs 6A, 6C and 7A). Interestingly, increasing frequencies of AM and SM B cells were not related to a global activation of memory responses to resident infectious agents within the host, as RhCMV-specific IgG antibody responses progressively declined over the 12 week period of acute infection (Fig 7B). Although plasma SIVmac251 gp130-specific IgG concentrations positively correlated with increasing frequencies of circulating SM (*P* < 0.0001) and AM (*P* < 0.0001) B cell subsets (Fig 7D), emerging SIV humoral responses were dysfunctional based on measurements of SIV p27-specific IgG avidity (Fig 7A). The absence of increasing SIV p27 antibody avidity over the first eight weeks of SIV infection differed strikingly from the increases in RhCMV-specific IgG avidity indexes that emerged over the first eight weeks of primary RhCMV infection experienced by these same macaques prior to infection with SIV (Fig 7C). Taken together, these observations including induction of SIV-specific IgG responses characterized by poor avidity, increases in circulating B cell counts, declining plasma RhCMV antibody concentrations, and increases in the SM and AM subsets coincident with decreases in the RM and naive B cell frequencies, were quite similar to B cell alterations reported for HIV-1 infection [5, 6, 47].

Analysis of humoral responses demonstrated by the single rapid SIV progressor revealed a persistent decline in frequency and absolute count of B cells in blood during the acute infection period (S3A Fig) that differed from the typical progressor infection (Fig 6A). Furthermore, changes in circulating B cell subsets included a progressive loss in SM and AM B cells and an increasing frequency of the RM subset within the SM population over the initial 8 weeks of SIV infection (S3B Fig). Even more dramatic changes within the SM subset were observed over weeks 11 − 19 after infection, with emergence of unconventional CD21^+^CD27^lo/-^ RM cell subset. Moreover, a CD21^-^CD27^-^ subpopulation was observed within the SM B cells of this rapid progressor at a later stage of SIV infection, representative of the DN or TLM subset observed in elderly humans [39, 48] and chronic HIV-1 infection [6, 40, 49, 50] (S3B Fig). These marked changes in B cell subsets were associated with a failure to develop detectable plasma IgG specific for either SIV gp130 or p27, while no changes were observed for RhCMV-specific antibody concentrations in plasma over this same time period of acute infection (S3C Fig). The progressive replacement of the circulating SM subset represented by both RM and AM populations with a DN memory subset, referred to as TLM by some reports, combined with a failure to develop SIV-specific antibodies, suggested dramatic changes in systemic B cell compartments associated with a significant deficit for induction of *de novo* B cell responses that merit future investigation.

## Discussion

A review of reports describing changes in B cell subset distribution in human disease and aging revealed significant variation in the combinations of memory markers including IgD, CD27, CD24, CD38, CD10, and CD21 used for B cell analysis [10, 24, 25, 37, 46, 51]. Reports describing circulating B cell subsets in rhesus macaques have been limited and typically based on a combination of either CD27 and CD21, or CD27 and IgD for characterizing memory B cell populations defined initially by CD20 or occasionally by CD19 staining [17–19, 22]. Accordingly, establishment of a phenotyping panel based on a combination of IgD, CD27, and CD21 as described herein, permitted separation of macaque B cells into SM, UM, and naïve subsets and further separation of the SM subset into AM and RM populations to allow a superior characterization of memory B cell subsets in rhesus macaques. Establishment and use of this expanded staining panel for circulating B cells also revealed a distinctive CD21^-^CD27^+^ UM subset that has not been recognized in macaques or reported for circulating human B cells. Future studies are warranted to determine if any functional differences exist between CD21^-^CD27^+^ and CD21^+^CD27^+^ UM subsets identified by this analysis.

Furthermore, analysis of circulating B cells revealed key differences in the frequencies of UM versus naïve subsets as well as AM versus RM subsets between macaques and humans. The prominent CD21^-^CD27^+^ UM B cell population observed in macaques may be a contributing factor for the greater frequency of UM B cells and lower frequency of circulating naïve B cells observed in young macaques compared to human donors. In agreement with our findings, multiple reports have previously described significantly lower frequencies of circulating naïve B cells (CD27^-^ B cells) in macaques (rhesus and cynomolgus) compared to frequencies of CD27^+^ B cells [16, 18, 20, 21, 52–54]. Our findings of higher frequencies of AM compared to RM subsets in macaques compared to human donors are also supported by results of other studies comparing humans and macaques [16, 18, 21]. These findings overall suggest a potentially important difference in B cell memory subset distribution in blood between rhesus macaques and humans. However, further consideration must be given to the role of variation in environment, social interactions, and chronic infections unique to captive rhesus macaque populations when comparing B cell subsets between macaque cohorts and human populations. Although the SPF macaque cohort tested in our studies is defined as free of simian retroviruses and specific rhesus herpes viruses (B virus, RhCMV) [55], this macaque cohort may still harbor other chronic infections including rhesus rhadinovirus (RRV), simian varicella virus (SVV), rhesus lymphocryptovirus (RLV), and rhesus papillomavirus (RhPV) that may also impact circulating B cell populations. Therefore, comparisons of circulating memory B cell subset distribution between humans and captive nonhuman primates will need to consider differences in environmental factors and host viromes in future studies.

Use of an expanded B cell staining panel revealed specific changes in multiple circulating B cell subsets that were apparent for a SPF rhesus macaque cohort transitioning from one to 2 − 5 years of age. A reduced frequency of the naïve B cell subset was matched with increased frequencies of UM, SM and AM subsets most likely associated with increasing numbers of antigen-experienced cells as the host transitioned from a young juvenile to a young adult. Similar trends in B cell subsets were observed for a second group of non-SPF macaques examined over the same age range but housed within a different primate center and tested by a more traditional B cell staining panel based on CD20, CD27, and IgD [38]. However, analysis of RM and AM subsets was not possible for this second cohort due to a more restricted staining panel excluding CD21. These findings for changes in naïve and memory B cell subsets in macaques transitioning from young juveniles to young adults agree with a recent report characterizing B cell subsets in rhesus macaques [35] and in a human cohort [46] over similar age ranges. These same findings, however, conflict with results from a second report testing similarly aged SPF and non-SPF rhesus macaques [19]. Interestingly, our data showed that changes in B cell subset distribution that emerge during the transition from young juvenile to young adult in both SPF and non-SPF macaque cohorts, are reversed in non-SPF macaques aging from young adults to considerably older adults between 25 to 30 years of age. Changes over this age span included very modest but significant increases in the naïve and DN B cell subsets and similarly very modest decreases in SM and UM subsets. These changes directly contrasted with transformations in circulating B cell subset frequencies described for macaques transitioning from adults to aged adults in a previous report [35]. Similarly, multiple reports describing B cell subset changes in aging human adults [12, 37, 46, 56, 57] reveal conflicting data regarding different human cohorts. However, our findings for changes in naïve, UM, SM and DN subsets in aging rhesus macaques agree with some human studies [34, 39, 56, 58]. In addition, the progressive reduction in circulating B cell numbers and frequency as observed in our aging macaque cohort, has been a consistent finding in human studies [11, 48, 59] and was previously reported for a different rhesus macaque cohort [35]. Importantly, comparisons between other studies of circulating memory B cell subsets in rhesus macaques and humans are confounded by differences in B cell staining panels, flow cytometry analysis, imperfect matching of age groups, and possible differences in environmental factors between different research primate centers, as well as between NHP and human cohorts. These considerations taken together with our findings for the impact of aging on circulating B cell subset distribution in captive rhesus macaques, still suggest that NHP animal models are appropriate for investigation of immunosenescence of B cells. Moreover, these NHP models may be valuable for distinguishing the effects of aging versus effects of life-long chronic infections such as herpesvirus infections that have been reported to impact B cell subsets and numbers [2–4, 19]. The issue of RhCMV infection in particular was not addressed in studies presented herein but is a focus of ongoing studies in our laboratory. Collectively, these results suggest the need for future studies using standardized B cell staining panels in conjunction with assessment for B cell function and testing of different tissue B cell subsets to further characterize rhesus macaque B cell immunosenescence as a model for human B cell aging.

Specific changes in circulating B cell subsets associated with a hypergammaglobulinemia, polyclonal activation, lymph node germinal center hyperplasia, and altered antibody responses to HIV-1 and other pathogens have been described in multiple reports for HIV-1 infection [5–8, 60]. Specific B cell alterations observed following SIVmac infection in our study included a precipitous reduction in frequency and absolute number of circulating B cells followed by a recovery within four weeks of infection; reduced frequencies of naïve, UM and RM B cell subsets; and increased frequencies of SM and AM B cell subsets. These same B cell alterations have previously been reported for SIV [21, 23, 52, 61] and HIV-1 [5–9]. Unique findings from our study revealed significant correlations between increases in circulating SM and AM subsets with emergence of SIV Env antibodies during acute infection. In addition, changes in B cell memory subsets and increases in B cell numbers were also associated with increasing SIV capsid antibody concentrations in plasma, humoral dysfunction including impaired development of SIV capsid antibody avidity in the face of increasing SIV capsid antibody titers, and reduced circulating RhCMV antibodies. Notably, our observations for the first twelve weeks of SIV infection do not show complete agreement with all reports describing B cell subset changes characteristic of typical SIV-related disease progression. One previous report described changes in AM and RM subsets during acute SIV infection along with emerging SIV Env antibody induction similar to our findings, but revealed no changes in pre-existing antibody titer to bacterial antigen flagellin (FliC) [21]. Also, the magnitudes of B cell count rebound and changes in memory subsets during acute SIV infection noted in other reports [20, 52] differed from our observations. These differences may be partially related to the use of different B cell staining panels that limit delineation of memory subsets, different functional assessments, and a restriction of time points tested during early and early chronic SIV infection by previous reports. Limitations within our studies included assessment of circulating B cells only and the absence of activation markers in our expanded B cell staining panel that have been included in other reports. Therefore, data generated from our studies cannot be directly compared to all previous reports of SIV-related alterations of humoral responses. Further characterization of SIV infection as an animal model for humoral responses to HIV-1 infection will require standardization of assays for identification of key B cell subsets and assessment of B cell function.

Interestingly, our findings for a single animal showing rapid disease progression during acute SIV infection are quite similar to changes in humoral responses previously reported for SIV rapid progressors. A progressive decline in circulating B cell counts was observed along with a reduced SM B cell frequency, further characterized by a progressive loss of the AM subset [21, 61] and associated with increasing frequencies of IgD^+^ and DN B cell subsets. Similar changes in the DN B cell subset were frequently reported for HIV-1 infection [6, 40, 49, 50], but rarely appreciated for accelerated SIV-induced disease in macaques [21]. Although alterations in pre-existing antibody responses to RhCMV were not detected in this single rapid progressor, antibody responses to SIV Gag and Env were not detectable within twelve weeks post infection. Severe restriction or absence of SIV-specific antibodies has been consistently reported for macaques showing rapid SIV and SHIV disease progression [21, 61–65], and also reported for HIV-1-infected humans showing rapid disease progression [66, 67]. Recent reports for the SIV animal model suggest possible mechanisms for the severe persistent B cell depletion and restricted SIV-specific antibody responses that include significant B cell activation characterized by increased B cell expression of PD-1 [21] and dysregulated germinal center (GC) formation related to a reduction or absence of IL-21 production in lymph nodes [61]. Taken together, both typical and rapid progressor phenotypes observed in our SIV-infected macaque cohort provide additional evidence that SIV disease progression is associated with specific alterations of circulating B cell subsets and deficits in humoral responses that are similar to those observed in HIV-1-infected patients and therefore warrant further investigation. Such mechanistic studies may identify potential targets for immune modulation leading to restoration of effective antiviral humoral responses in HIV-1-infected cohorts for which ineffective neutralizing antibody responses remain a hurdle for virus elimination from the host.

Characterization of circulating B cell populations in healthy and aging rhesus macaque populations will be critical for effective utilization of this valuable animal model to investigate humoral immune alterations associated with human immunosenescence and infectious diseases. The initial steps to achieve this goal involved development of an expanded multicolor flow cytometric panel using B cell markers that would define multiple memory B cell subsets significant in human aging and disease and facilitate a comparison of macaque and human B cell subset distribution in health. Our studies revealed changes in distribution of naïve, UM and SM B cell subsets including RM and AM subpopulations in SPF macaques during the first five years of life. Examination of a large non-SPF macaque cohort spanning 30 years of age with a more standard but less stringent flow cytometric B cell staining panel based on IgD and CD27, also revealed similar changes in naïve and memory B cell subsets during the same age range. However, trends for naïve, UM, and SM subset frequencies reversed over ages spanning 6 to 30 years, revealing changes that were both similar and different compared to changes reported for circulating B cell subsets in aging human populations. Lastly, this expanded B cell staining panel was utilized to examine changes in memory B cell subsets associated with functional alterations of the humoral system during acute SIVmac251 infection. This investigation of acute SIV infection revealed trends in circulating B cell memory subset distribution associated with unique antibody response patterns that proved specific to different disease progression phenotypes. Changes in B cell memory populations were comparable to those reported for HIV infection and therefore confirmed the utility of this particular B cell marker combination for multicolor flow cytometric analysis of circulating B cell subsets in the rhesus macaque animal model for HIV. Accordingly, these observations provide further evidence of the utility of the NHP model for investigation of mechanisms responsible for B cell subset alterations and humoral response deficits observed with HIV-1 infection. These findings also suggest possible opportunities afforded by the NHP model for examination of B cell changes associated with other infectious agents including herpesviruses associated with life-long persistent infections in the human host.

## Supporting Information

S1 Fig Identification of four phenotypically distinct B cell subsets in the peripheral blood of healthy human donors by CD20, CD21, and CD27. (**A**) Shown are representative FACS plots for identifying B cell subsets by surface expression of CD21 and CD27: tissue-like memory (TLM; CD21^lo/-^CD27^-^), activated memory (AM; CD21^-^CD27^+^), resting memory (RM; CD21^+^CD27^+^), and naïve (CD21^+^CD27^-^). (**B**) Frequencies of B cell subsets were determined by this gating strategy in peripheral blood of healthy human adults (*n* = 12). Bars in each graph indicate median frequencies.
(PDF)

S2 Fig Identification of four phenotypically distinct B cell subsets in the peripheral blood of healthy SPF rhesus macaques by CD20, IgD, and CD27. (**A**) Shown are representative FACS plots of a gating strategy used to identify macaque B cell subsets by surface expression of IgD and CD27: switched memory (SM; IgD^-^CD27^+^), unswitched memory (UM; IgD^+^CD27^+^), naïve (IgD^+^CD27^-^); and double negative (IgD^-^CD27^-^). (**B**) Frequencies of B cell subsets were determined by this gating strategy in peripheral blood of healthy SPF rhesus macaques (age 1 yr, *n* = 23; age 2 − 5 yrs, *n* = 21). Statistical analyses between two age cohorts of SPF macaques were performed using nonparametric Mann-Whitney tests. Symbol: *** *P* < 0.001; **** *P* < 0.0001.
(TIF)

S3 Fig Impact of SIV infection on distribution of B cell subset in peripheral blood and induction of antiviral antibody response in a rapid progressor rhesus macaque. (**A**) Measurement of viral loads, B and CD4^+^ T cell frequencies and counts following SIV infection are shown. (**B**) FACS plots depict the progressive shift of circulating B cell subsets over the duration of SIV infection. (**C**) Plasma IgG titers of anti-SIV gp130, SIV p27, and RhCMV virions during the course of acute-early chronic SIV infection are shown.
(TIF)

S1 Table Age breakdown summary of macaque subjects used in this study. (TIF)

S2 Table Summary of all human subjects used in this study. (PDF)
