# Supplementary material for: Changes in Circulating B Cell Subsets Associated with Aging and Acute SIV Infection in Rhesus Macaques
Source: PLoS One. 2017 Jan 17;12(1):e0170154. doi: 10.1371/journal.pone.0170154 (PMC5240950; doi:10.1371/journal.pone.0170154)
Supplement: S1 Fig — (A) Shown are representative FACS plots for identifying B cell subsets by surface expression of CD21 and CD27: tissue-like memory (TLM; CD21lo/-CD27-), activated memory (AM; CD21-CD27+), resting memory (RM; CD21+CD27+), and naïve (CD21+CD27-). (B) Frequencies of B cell subsets were determined by this gating strategy in peripheral blood of healthy human adults (n = 12). Bars in each graph indicate median frequencies. (PDF) [file pone.0170154.s001.pdf]

S1 Table. Age breakdown summary of macaque subjects used in this study.

	Age	Sample number
**SPF macaque cohort in CNPRC (44 total)**		
	1	23
	2	6
	3	9
	4	3
	5	3
**Non-SPF macaque cohort in ONPRC (275 total)**		
	2	19
	3	6
	4	8
	5	6
	6	11
	7	11
	8	13
	9	15
	10	16
	11	10
	12	18
	13	2
	14	13
	15	6
	16	2
	17	5
	18	30
	19	35
	20	13
	21	10
	22	2
	23	7
	24	9
	25	3
	27	1
	28	1
	29	2
	30	1
