# Supplementary figures and images for: Changes in Circulating B Cell Subsets Associated with Aging and Acute SIV Infection in Rhesus Macaques
Source: PLoS One. 2017 Jan 17;12(1):e0170154. doi: 10.1371/journal.pone.0170154 (PMC5240950; doi:10.1371/journal.pone.0170154)

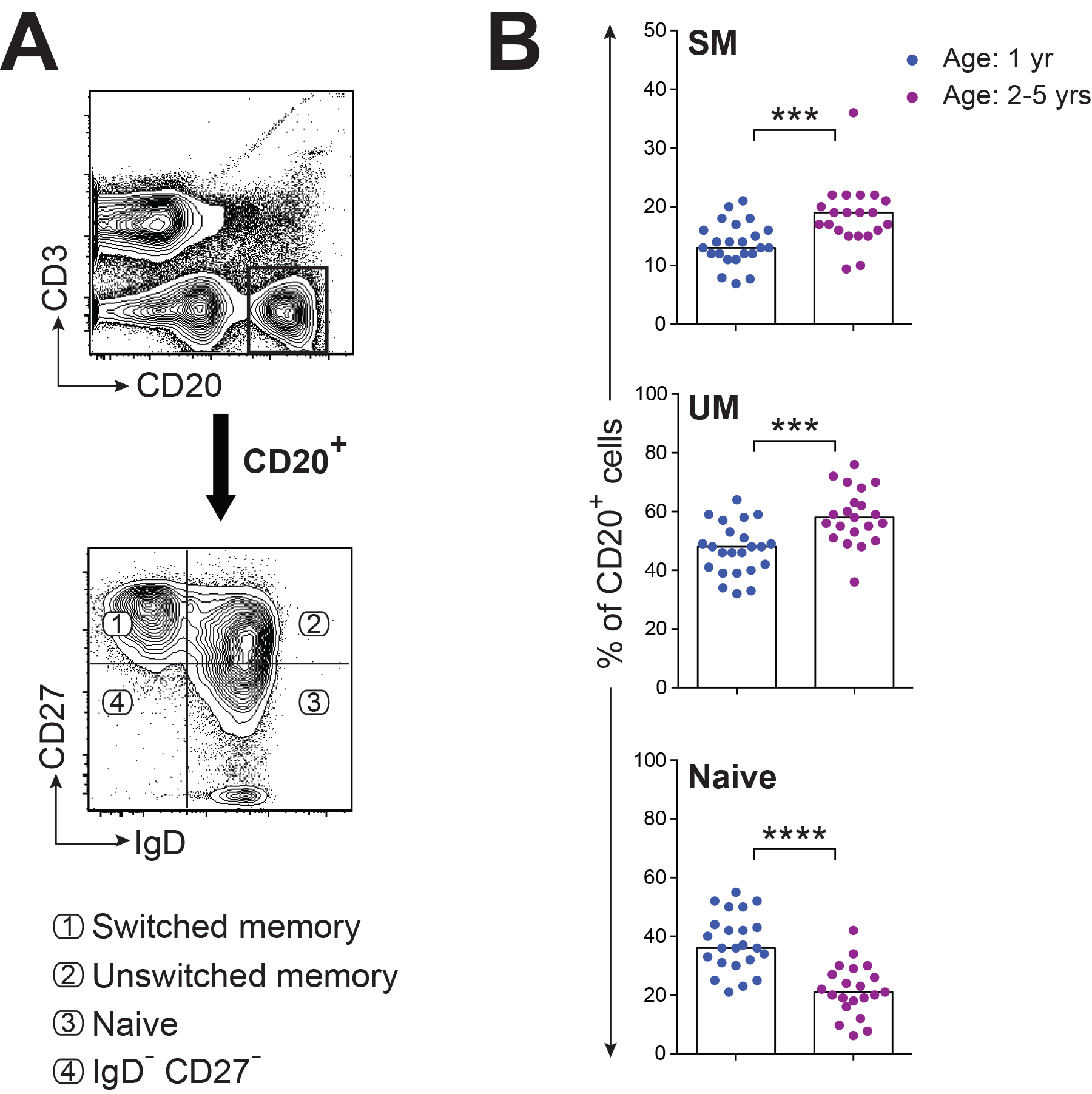

Supplement: S2 Fig — (A) Shown are representative FACS plots of a gating strategy used to identify macaque B cell subsets by surface expression of IgD and CD27: switched memory (SM; IgD-CD27+), unswitched memory (UM; IgD+CD27+), naïve (IgD+CD27-); and double negative (IgD-CD27-). (B) Frequencies of B cell subsets were determined by this gating strategy in peripheral blood of healthy SPF rhesus macaques (age 1 yr, n = 23; age 2 − 5 yrs, n = 21). Statistical analyses between two age cohorts of SPF macaques were performed using nonparametric Mann-Whitney tests. Symbol: *** P < 0.001; **** P < 0.0001. (TIF) [file pone.0170154.s002.tif]

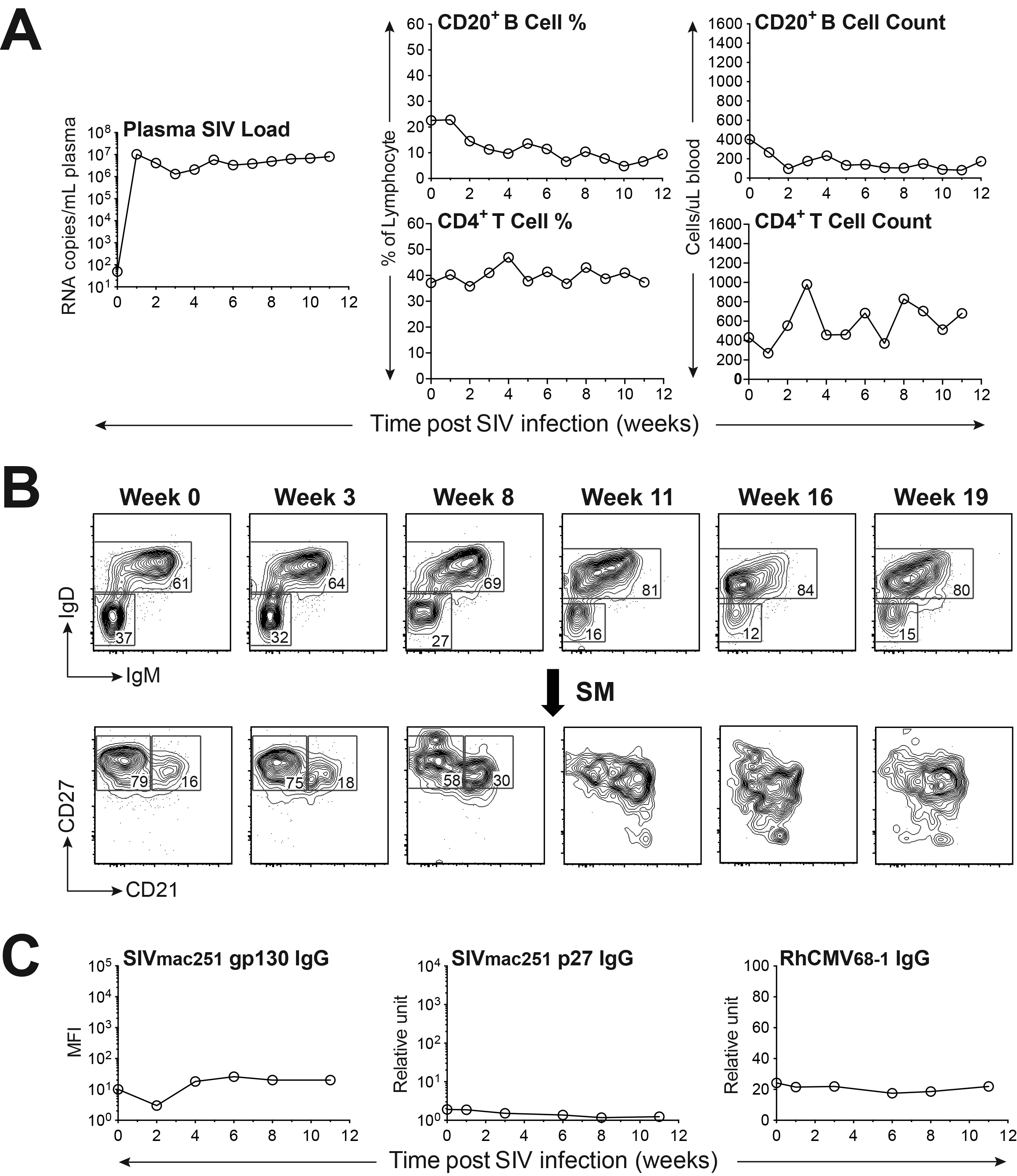

Supplement: S3 Fig — (A) Measurement of viral loads, B and CD4+ T cell frequencies and counts following SIV infection are shown. (B) FACS plots depict the progressive shift of circulating B cell subsets over the duration of SIV infection. (C) Plasma IgG titers of anti-SIV gp130, SIV p27, and RhCMV virions during the course of acute-early chronic SIV infection are shown. (TIF) [file pone.0170154.s003.tif]

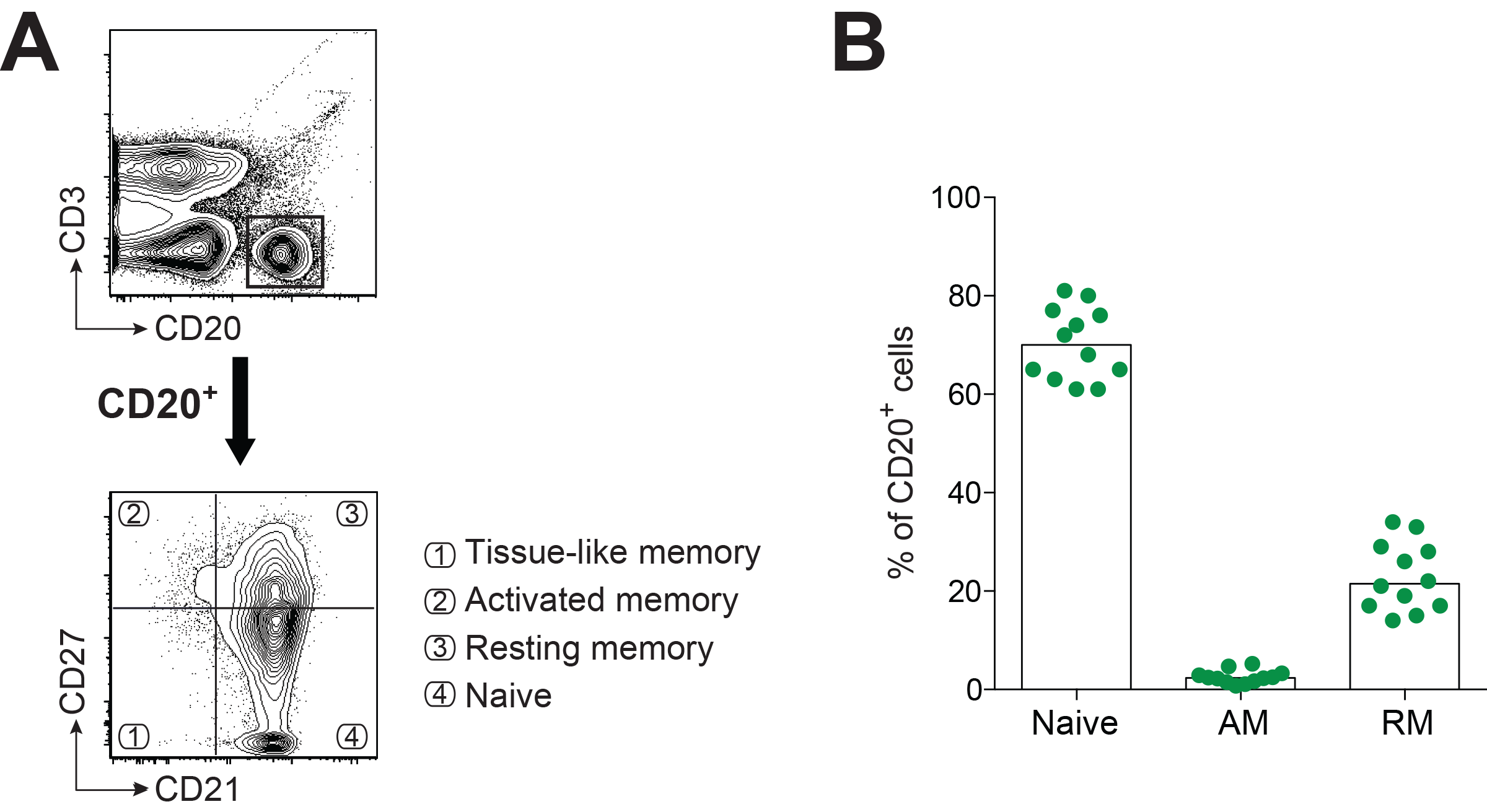

Supplement: S1 Table — (TIF) [file pone.0170154.s004.tif]
