# Supplementary material for: Changes in Circulating B Cell Subsets Associated with Aging and Acute SIV Infection in Rhesus Macaques
Source: PLoS One. 2017 Jan 17;12(1):e0170154. doi: 10.1371/journal.pone.0170154 (PMC5240950; doi:10.1371/journal.pone.0170154)
Supplement: S2 Table — (PDF) [file pone.0170154.s005.pdf]

S2 Table. Summary of all human subjects used in this study.

	Age	Gender	Blood type	CMV status
**Human donor (12 total)**				
	26	M	A+	N
	35	M	O+	P
	41	M	B+	N
	44	M	A+	N
	47	M	A+	P
	47	M	A+	N
	51	F	O+	N
	54	F	A+	N
	55	M	B+	N
	56	M	O+	N
	63	M	A+	N
	68	M	A+	N

M: male
F: female
P: positive
N: negative
